# Bulk and interface engineering of 1.7 eV–bandgap chalcogenide solar cells enabling record efficiency

**DOI:** 10.1126/sciadv.aed4703

**Published:** 2026-03-11

**Authors:** Shogo Ishizuka, Noboru Taguchi

**Affiliations:** ^1^ Renewable Energy Advanced Research Center, National Institute of Advanced Industrial Science and Technology, 1-1-1 Umezono, Tsukuba, Ibaraki 305-8568, Japan.; ^2^ Research Institute of Electrochemical Energy, National Institute of Advanced Industrial Science and Technology, 1-8-31 Midorigaoka, Ikeda, Osaka 563-8577, Japan.

## Abstract

Wide-bandgap chalcogenide photovoltaics offer strong potential for tandem solar cells and solar-driven hydrogen generation via water splitting, yet their performance remains limited by persistent interfacial and bulk defects. Here, we demonstrate enhanced efficiency in 1.7–electron volt CuGaSe_2_ thin-film solar cells through aluminum (Al) alloying and rubidium (Rb) incorporation. The Al- and Rb-modified CuGaSe_2_ absorber exhibits fundamentally distinct interfacial chemistry, structural properties, and metastable defect behavior compared with conventional narrow-bandgap Cu (In,Ga)Se_2_ solar cells. Moreover, we demonstrate that introducing a steep Al concentration gradient to engineer a back-surface electric field is a highly effective means of boosting device performance, even at an Al content of approximately 1 atomic % or less. By integrating these strategies, we achieve a higher open-circuit voltage without compromising photovoltaic efficiency, establishing a performance benchmark for wide-bandgap (1.65 to 1.75 electron volts) chalcogenides. These findings highlight the unique characteristics of wide-bandgap chalcopyrites and suggest a promising pathway toward next-generation, high-efficiency photovoltaic technologies.

## Teaser

Bulk and interface engineering strategies are developed to improve the performance of wide-bandgap chalcogenide solar cells.

## INTRODUCTION

Chalcogenides, including II–VI compounds and their derivatives such as CdTe, CuInSe_2_, and Cu_2_ZnSnS_4_, are widely recognized as promising thin-film photovoltaic absorbers with high power conversion efficiencies (PCE) (*1*–*4*). In recent years, the focus has intensified on single-junction solar cells as well as tandem (multijunction) solar cells that use a wide variety of materials such as silicon, metal-halide perovskite, and other compound semiconductors (*5*–*7*). Chalcopyrite CuInSe_2_–based materials, which have a relatively narrow bandgap energy (*E*_g_) of approximately 1.0 to 1.2 eV, are considered promising absorbers for bottom cells (*6*–*8*). Conversely, cost-effective top-cell materials are required to realize high-performance tandem solar cells suitable for civilian use. However, in contrast to narrow-bandgap materials such as silicon and CuInSe_2_-based compounds, a wide-bandgap top cell that simultaneously satisfies all three key requirements—high PCE, good stability, and cost-effectiveness—has yet to be realized, even among metal-halide perovskites and III–V compound semiconductors.

Nevertheless, chalcogenide compounds remain compelling candidates for wide-bandgap top cells. Their potential has been highlighted by the proven success of narrow-bandgap single-junction solar cells in practical applications; however, their wide-bandgap counterparts lack establishment and remain underexplored. CuInSe_2_-based materials provide broad bandgap tunability via compositional variations. Bandgap widening is accomplished through increasing the gallium content (*9*) and/or sulfur alloying (*10*). Ternary CuGaSe_2_, with an *E*_g_ of approximately 1.7 eV, is an attractive starting material for further development. Such wide-bandgap photoabsorber materials are also attractive for diverse applications in energy-conversion devices, including photocathodes for hydrogen generation in photoelectrochemical water-splitting cells (*11*, *12*).

Along with the mainstream narrow-bandgap CuInSe_2_-based solar cells, wide-bandgap CuGaSe_2_ solar cells have followed a distinct research path. A single-crystalline CuGaSe_2_ photoabsorber layer yielded 5% PCE with a CdS film deposited by the coevaporation of elemental Cd and S sources in 1977 (*13*). After the report of a PCE of 9.7% demonstrated for a single-crystalline CuGaSe_2_ absorber layer in 1997 (*14*), a polycrystalline CuGaSe_2_ thin-film absorber exhibited a comparable efficiency of 9.5%, as an independently certified value in 2003 (*15*). In 2013, double-digit (≥10%) efficiency was initially achieved in CuGaSe_2_ solar cells (*16*, *17*). In 2017, improvements in open circuit voltage (*V*_OC_) exceeding 1 V and a concomitant PCE enhancement (11.9%) were demonstrated using a ZnSnO buffer layer, which was used instead of a CdS buffer (*18*). The beneficial effects of the addition of alkali metals and silver alloying on CuGaSe_2_ solar cells have also been reported (*19*), although the effects observed for In-free devices somewhat differ from those observed for In-containing Cu(In,Ga)Se_2_ (CIGS) devices.

Recently, we developed a method that enhances the performance of CuGaSe_2_ solar cells by incorporating aluminum (*20*, *21*). The formation of a [Al]/{[Al] + [Ga]} compositional gradient, along with the concomitant development of a back surface field (BSF) or a functionally equivalent structure, enhanced carrier transport and device performance, leading to an independently certified PCE exceeding 12% [PCE: 12.25%, *V*_OC_: 0.959 V, short-circuit current density (*J*_SC_): 17.64 mA cm^−2^, fill factor (FF): 72.5%, cell area: 0.499 cm^2^] (*21*). Furthermore, a *V*_OC_ exceeding 1 V (PCE: 11.7%, *V*_OC_: 1.007 V, *J*_SC_: 15.62 mA cm^−2^, FF: 74.5%, cell area: 0.5076 cm^2^) was achieved by using a high Al content despite the use of a conventional CdS buffer layer, although the PCE remained below 12% (*21*).

Because improvements in the interface and bulk quality of CuGaSe_2_ solar cell devices are essential for further development, we analyzed the chemical and physical interface structures of the high–Al-content CuGaSe_2_ device exhibiting a *V*_OC_ of 1 V. The enhancement in the photovoltaic performance attributed to the formation of metastable acceptors, proposed to originate from selenium and copper double-vacancy complexes (*V*_Se_–*V*_Cu_), is termed the light-soaking effect in CuInSe_2_-based solar cells (*22*–*25*). This increases the hole carrier density and thereby improves device performance, such as an enhanced *V*_OC_; this effect is well known in CIGS solar cells. In the present study, we examined the light-soaking effect in CuGaSe_2_-based devices. Last, we present our recent progress and prospects for wide-bandgap chalcopyrite solar cells, featuring a high *V*_OC_ of approximately 1 V, together with a PCE greater than 12%.

## RESULTS

### Interface analysis of high-*V*_OC_ CuGaSe_2_:Al,RbF/CdS solar cells

In this section, we examine the nano- and microstructural features of a high-*V*_OC_ CuGaSe_2_ device containing Al and Rb. A large *V*_OC_ deficit (Δ*V*_OC_), which is estimated as the difference between *E*_g_/*q* and the measured *V*_OC_, where *q* is the electron charge, remains a long-standing obstacle for wide-bandgap chalcopyrite. Narrow-bandgap CIGS devices typically exhibit Δ*V*_OC_ values of 0.3 to 0.4 V (*26*), whereas CuGaSe_2_ devices exhibit values near 0.8 V, with most reported *V*_OC_ approximately 0.9 V or less (*27*). Only a few studies have achieved *V*_OC_ above 1 V in CuGaSe_2_ (*18*, *21*), including our prior work (*19*), using an Al-alloyed, Rb-incorporated CuGaSe_2_ photoabsorber (CuGaSe_2_:Al,RbF) with a CdS buffer. Thus, to gain further insight into the factors underlying the performance of high-*V*_OC_ CuGaSe_2_ devices, we analyzed key device characteristics. The CuGaSe_2_ absorber was grown using a three-stage process, with Al and RbF supplied during the early first stage and additional RbF introduced during the final part of the third stage. Rb was incorporated through RbF addition; however, fluorine is not retained after growth (*28*); therefore, “CuGaSe_2_:Al,RbF” simply denotes the use of Al and RbF as source materials.

**Fig. 1.**
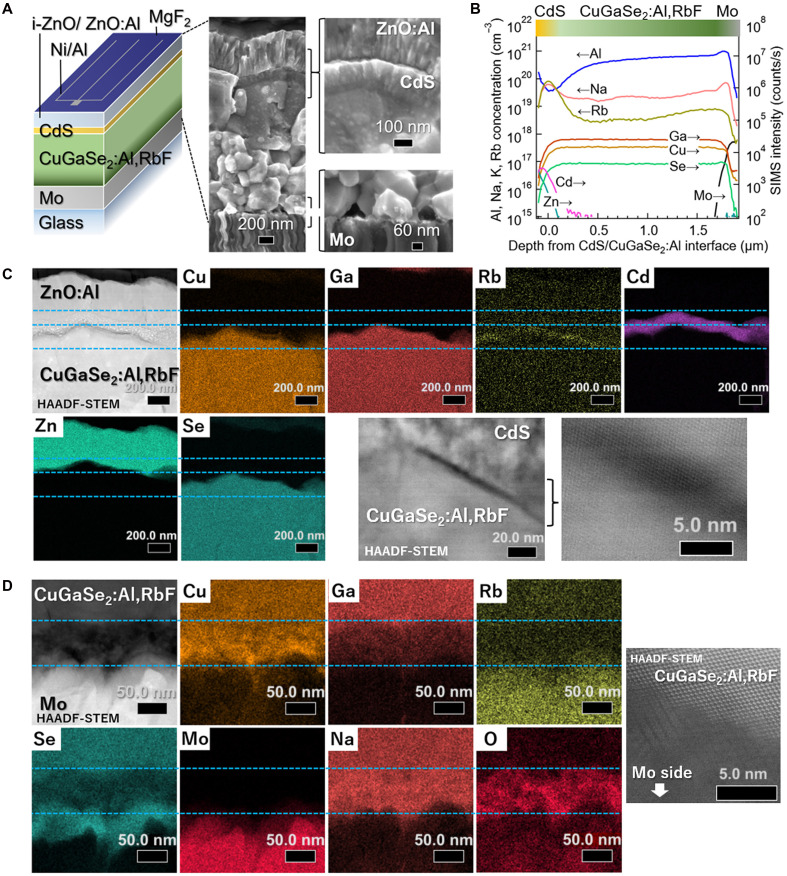
Structure of a high-*V*_OC_ CuGaSe_2_:Al,RbF solar cell device. (**A**) Schematic of the CuGaSe_2_:Al,RbF solar cell device structure and cross-sectional SEM images. (**B**) SIMS depth profiles of the CuGaSe_2_:Al,RbF photoabsorber layer. (**C**) TEM-EDX results and high-magnification HAADF-STEM images of the CuGaSe_2_:Al,RbF/CdS front interface and (**D**) the Mo/CuGaSe_2_:Al,RbF rear interface. Blue dotted lines are provided as a guide to the eye.

The cross-sectional scanning electron microscopy (SEM) images, secondary ion mass spectrometry (SIMS) profiles, and scanning transmission electron microscopy combined with energy-dispersive x-ray spectroscopy (TEM-EDX) results obtained from the high-*V*_OC_ CuGaSe_2_:Al,RbF device are shown in Fig. 1 (A to D). These samples, from the same growth batch as our earlier *V*_OC_ ~1.0 V devices with PCE >11% (*21*), show grain sizes of a few hundred nanometers with numerous voids at the Mo/CuGaSe_2_:Al,RbF interface. The SIMS depth profiling reveals an Al concentration gradient, whereas Cu, Ga, and Se distributions remain uniform (Fig. 1B). Strictly speaking, elemental Ga exhibited a slight compositional gradient in the direction opposite to that of the Al distribution (fig. S1A). Al concentrations are 4 × 10^19^ cm^−3^ near the CdS interface [0.06 atomic % (at %)] and 8 × 10^20^ cm^−3^ near the Mo interface (1.25 at %). Adding several tens of percent Ga to CuInSe_2_ reduces the grain size, and a similar phenomenon is observed when Al is added to CuInSe_2_ (*20*). A similar effect is expected with the incorporation of Al into CuGaSe_2_. However, the Al content of the film is approximately 1 at % or less and is therefore unlikely to have a substantial impact on the grain size. When CuGaSe_2_ films are grown on Mo-coated soda-lime glass (SLG) substrates at substrate temperatures (*T*_S_) comparable to those typically used for CuInSe_2_ or CIGS (500° to 550°C), their grain size is inherently as small as that seen in Fig. 1A. This trend is well recognized in the three-stage coevaporative growth of polycrystalline chalcopyrite films (*9*).

TEM-EDX results for the CuGaSe_2_:Al,RbF/CdS and Mo/CuGaSe_2_:Al,RbF interfaces are shown in Fig. 1 (C and D), respectively. The elemental Al signal (Kα: 1.49 keV) overlaps with that of Se (Lβ: 1.42 keV); therefore, the EDX map of Al is not shown. Instead, its distribution is revealed by the SIMS profile shown in Fig. 1B. For CIGS, heavy alkali fluorides (KF, RbF, and CsF) applied as postdeposition treatments (PDTs) enhance photovoltaic performance (*29*, *30*). Although three-stage processed CIGS films inherently exhibit relatively Cu-depleted surfaces, PDT has been reported to increase Cu depletion (*29*). In addition, PDT often induces the formation of alkali-In-Se compounds such as RbInSe_2_ on the film surface (*31*, *32*). However, such phases do not form on In-free CuGaSe_2_ after PDT (*31*, *32*), and the formation of distinct RbGaSe_2_ phase is not observed in our coevaporated CuGaSe_2_:Al,RbF films, although the EDX maps reveal Rb accumulation in the Cu-depleted surface region (Fig. 1C). Magnified EDX maps of elemental Cu, Ga, and Rb are shown in fig. S1B. The formation of such alkali metal–related secondary phases depends on the presence of elemental In and the alkali-metal species used. Our earlier study found no formation of CsInSe_2_ phases after CsF-PDT on CIGS, despite performance improvements (*33*), indicating that the crystallization of alkali-In-Se phases is not essential. Even when present as impurities introduced through doping, they may improve device performance in the bulk or at grain boundaries.

The TEM-EDX results for the Mo/CuGaSe_2_:Al,RbF rear interface are shown in Fig. 1D. Voids and interstices are often observed at the rear interface, regardless of the absorber material (CuInSe_2_ or CuGaSe_2_) or the grain size (*34*). These interstices may result not only from the sample preparation for cross-sectional observation but also from being present before it (Fig. 1A).

Strong Cu, Na, and O signals are detected at the rear interface (Fig. 1D). Before discussing the behavior of Cu at grain boundaries and interfaces, the following points should be noted. Both Cu deficiency and precipitation at the grain boundaries within CIGS thin films have been reported (*34*–*38*). Several film growth mechanisms, including the vapor-liquid-solid process (*39*) and topotactic process (*40*), which use Cu-rich phases involving Cu_2-x_Se secondary phases, have been proposed to obtain films with large grain sizes and high quality (low defect density). Although the final composition is adjusted to be slightly Cu-poor, the three-stage process is a method that uses this approach (*41*). Here, “Cu-rich” and “Cu-poor” indicate whether the [Cu]/[Group III] composition ratio (Cu/III) is greater or less than unity, respectively. In several cases, the grain boundaries within the CIGS films often show Cu-poor characteristics (*34*, *36*–*38*). However, Cu-rich behavior is frequently observed at random grain boundaries formed by misorientations between adjacent grains (*34*, *36*). In addition, when the growth temperature is low or the [Ga]/{[Ga] + [In]} ratio is high, the Cu_2-x_Se phases often remain in the film, even under Cu-poor conditions (*42*).

The Cu/III ratios of the film used in Fig. 1 (A to D) are 0.76 at the surface region and 0.98 in the bulk, were estimated by electron-probe microanalyzer (EPMA) measurements. Therefore, the Cu/III ratio of the film is typical of Cu-poor films prepared using a three-stage process. On the basis of our experience, Cu precipitation is observed during prolonged TEM observations at high acceleration voltages (e.g., 300 kV); therefore, this possibility cannot be entirely excluded. Nevertheless, the condition at the Mo/CuGaSe_2_:Al,RbF interface is considered to be closer to that of random grain boundaries rather than Σ3 grain boundaries within the bulk, where Cu-poor behavior is typically observed. Therefore, the Cu-rich behavior at the rear interface is not unexpected.

**Fig. 2.**
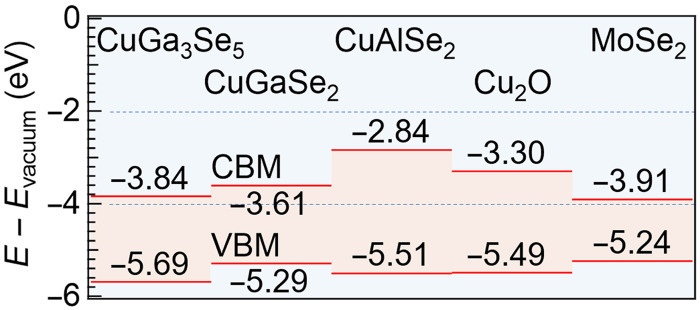
Energy band diagram. Correlative energy band diagram of CuGa_3_Se_5_, CuGaSe_2_, CuAlSe_2_, Cu_2_O, and MoSe_2_.

For the growth of the CuGaSe_2_:Al,RbF films, RbF is cosupplied with Al for approximately one-third of the first stage (*21*). In contrast to PDT, where Na is expelled and replaced by heavier alkali metals such as K or Rb (*29*, *30*), Rb is incorporated from the start of growth. This likely reduces Na incorporation, which originates from the SLG substrate and diffuses through the Mo back contact, into the Ga_2_Se_3_ precursor formed in the first stage. While Na and O are uncorrelated when NaF precursors are applied (*43*), their concentrations are strongly coupled when Na diffuses from the SLG substrate (*44*, *45*), a trend also observed in Fig. 1D. TEM-EDX results suggest that the rear interface could be described as Mo/Mo-Se (MoSe_2_)/Cu-Na-O/CuGaSe_2_:Al,RbF. High-angle annular dark field–scanning TEM (HAADF-STEM) reveal an amorphous or nanocrystalline interlayer beneath the CuGaSe_2_:Al,RbF absorber. MoSe_2_ is a well-known interfacial compound that provides favorable ohmic contact at Mo/CIGS interfaces (*46*–*48*). In contrast, the role of Cu-Na-O compounds remains unclear. Na-doped Cu_2_O (Cu_2_O:Na) is a p-type semiconductor with hole concentrations spanning 10^13^ to 10^19^ cm^−3^ (*49*), implying the possibility that similar Cu-Na-O phases at the rear interface influence the carrier transport. The energy band diagram (*27*, *50*–*55*) shown in Fig. 2 suggests that if such phases behave similarly to Cu_2_O, they can form barriers to both electrons and holes. However, Na doping into Cu_2_O lowers the Fermi level, resulting in upward band bending of both the conduction band minimum (CBM) and valence band maximum (VBM). In practice, because the Al concentration is extremely low (~1 at %), even if a Cu_2_O-like compound is present at the interface with MoSe_2_, the energy band alignment at the CuGaSe_2_:Al,RbF/Cu_2_O interface would be expected to be closer to that of CuGaSe_2_/Cu_2_O than to that of CuAlSe_2_/Cu_2_O. Consequently, this maintains hole transport while serving as an electron barrier. Although Al incorporation lowers the VBM, its concentration near the rear interface is only ~1 at %, making its influence negligible relative to CBM shifts. Notably, the CBM offset between CuAlSe_2_ and CuGaSe_2_ (0.77 eV) is significantly greater than the VBM offset (0.22 eV). Overall, these results would be more favorable than the presence of Cu-deficient phases such as CuGa_3_Se_5_ at the rear interface, which could impose significant hole barriers. Although the Cu_2_O:Na model is based on strong Cu, Na, and O signals, further studies are needed to confirm whether the interfacial phase is a distinct compound or a mixture that possibly includes Ga or Se. Other candidates, such as layered NaCuO_2_ (*56*–*59*) or Se-containing analogs, cannot be excluded. Nevertheless, a higher Cu content is expected to enhance *d*-orbital interactions, increase the VBM, and improve carrier transport. This explains why the device performance was sustained even in the presence of strong Cu, Na, and O signals at the rear interface.

### Metastable behaviors in CuGaSe_2_-based solar cells

The effectiveness of heat-light soaking (HLS) or bias soaking in enhancing CIGS solar cell performance has been widely reported (*60*–*63*). The mechanism is attributed to a metastable vacancy complex involving Se and Cu (*V*_Se_ + *V*_Cu_), which is activated under illumination to emit holes [(*V*_Se_ + *V*_Cu_)^+^ → (*V*_Se_ + *V*_Cu_)^−^ + 2 holes], thereby generating metastable acceptors (*23*). Experimental evidence supports this mechanism as the origin of the metastability (*25*). Thus, HLS improves the performance of CuInSe_2_ and CIGS devices. However, in our previous study, no significant improvement was observed for In-free CuGaSe_2_ devices (*31*). In the present work, we further found that the impact of the HLS on CuGaSe_2_ devices was influenced by the cell separation process, as discussed in the following section, whereas CIGS devices exhibited no such dependence (*64*).

**Fig. 3.**
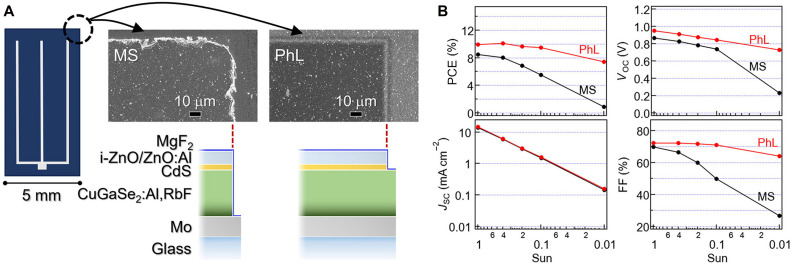
Effect of the cell edge on device performance. (**A**) Schematic and SEM images of MS- and PhL-processed cell edges. (**B**) Light intensity dependence of solar cell parameters for MS- and PhL-processed CuGaSe_2_:Al,RbF devices.

Two processes were compared: conventional mechanical scribing (MS), which removes the CuGaSe_2_:Al,RbF layer and above, and photolithography (PhL) followed by etching, which eliminates only CdS and above (Fig. 3A). An MgF_2_ antireflection coating (ARC) was applied after separation. These two processes strongly affect the shunt resistance (*R*_SH_); PhL yields markedly higher *R*_SH_, improving the FF (*64*). Previously, the performance gap between MS- and PhL-processed CIGS devices was minor under 1-sun illumination but was more pronounced under reduced illumination (*64*). MS devices degraded steeply, whereas PhL devices maintained or even improved the FF and PCE under low-sun conditions (*64*, *65*). In the present work, the CuGaSe_2_:Al,RbF devices showed similar behaviors (Fig. 3B) regardless of Al and Rb incorporation.

Nevertheless, CuGaSe_2_ and CIGS exhibited distinct *R*_SH_ characteristics. The diode parameters of the MS- and PhL-processed CuGaSe_2_ devices are summarized in table S1. The corresponding diode parameters for CIGS devices can be found in our previous study (*64*). These parameters were calculated from current-voltage curves using a single-diode model following Sites’ method (*66*). For the PhL-processed CuGaSe_2_:Al,RbF and CuGaSe_2_ devices, *R*_SH_ values under dark (~1 × 10^5^ Ω·cm^2^) and illumination (~2000 Ω·cm^2^) are comparable but significantly lower than those of PhL-CIGS (under dark: nominally infinite, under illumination: ~7000 Ω·cm^2^) (*64*). This result is likely attributable to factors that deteriorate the diode characteristics in CuGaSe_2_, including residual Cu_2-x_Se phases that may remain in the films, as well as intrinsic deep defect levels, in contrast to CIGS, as discussed later. In addition, CuGaSe_2_-based device properties under HLS are highly sensitive to cell-edge conditions, as discussed next.

All devices were MgF_2_-coated but not encapsulated with a barrier film or cover glass, as in commercial solar modules. Thus, the MS devices had exposed CuGaSe_2_ cross sections, whereas the PhL edges were almost entirely covered (fig. S2). For CIGS devices, HLS increased the nominal carrier density (*N*_CV_) and improved the device performance, regardless of the separation method (*64*). In contrast, this is not the case for In-free CuGaSe_2_ devices.

**Fig. 4.**
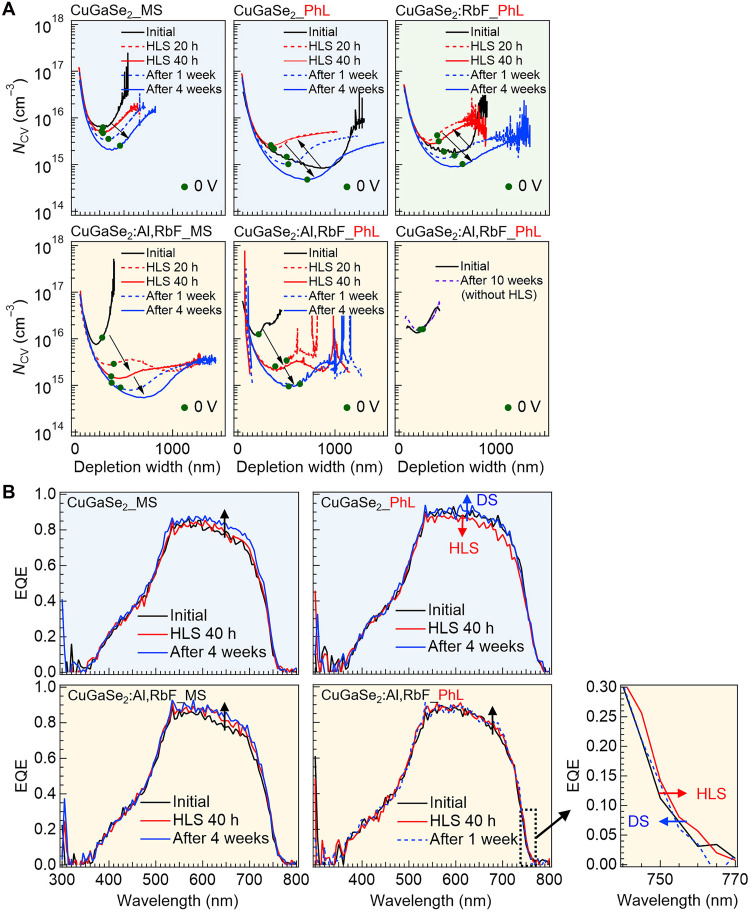
Effect of HLS on CuGaSe_2_-based devices. (**A**) Variations in *N*_CV_-depletion layer width curves and (**B**) corresponding EQE curves under HLS at 90°C and after its cessation. h, hours.

Figure 4A shows the typical variations in the *N*_CV_-depletion layer width curves derived from capacitance*-*voltage (*C*-*V*) measurements for the MS- and PhL-processed CuGaSe_2_ and CuGaSe_2_:Al,RbF devices. Measurements were performed before HLS (initial), after 20 and 40 hours of HLS at 90°C in a nitrogen-purged box, and after 1 and 4 weeks of dark storage (DS) at approximately 25°C. For comparison, the curves for a PhL-processed CuGaSe_2_:RbF device [Al-free, RbF supplied only at the end of the growth, corresponding to sample type #2 in our previous study (*21*)] and a CuGaSe_2_:Al,RbF device without HLS are included. The *N*_CV_ of the MS-CuGaSe_2_ device decreases upon HLS treatment, which is consistent with our previous results (*31*). *N*_CV_ continues to decline even after the cessation of HLS. In contrast, the PhL-CuGaSe_2_ device shows an initial *N*_CV_ increase during HLS, followed by a gradual decrease after HLS cessation. A similar trend is observed in the Al-free CuGaSe_2_:RbF device. On the other hand, CuGaSe_2_:Al,RbF devices exhibited only decreases in *N*_CV_ upon HLS at 90°C, irrespective of the separation method. No such time-dependent changes are observed in the absence of HLS.

According to the *V*_Se_ + *V*_Cu_ vacancy complex model, the metastable acceptor mechanism should be the same in CuInSe_2_ and CuGaSe_2_ (*23*), implying that *N*_CV_ should increase upon HLS. In CIGS, HLS at 90° to 130°C enhances performance (*60*–*62*). While Fig. 4 (A and B) shows results at 90°C, we also examined 50°C HLS (fig. S3A). At this lower temperature, *N*_CV_ increases in the CuGaSe_2_:RbF devices regardless of the separation method, which is consistent with the PhL-processed Al-free CuGaSe_2_ and CuGaSe_2_:RbF (Fig. 4A) and with CIGS. These results suggest that CuGaSe_2_ is more temperature-sensitive than CIGS (*31*, *67*, *68*) and that high-temperature HLS induces distinct detrimental defects that compensate for metastable acceptors and suppress the expected *N*_CV_ increase.

The corresponding external quantum efficiency (EQE) curves are shown in Fig. 4B. Variations in the depletion width are directly reflected in photocurrent generation. In CIGS, HLS often causes a redshift of the absorption edge (*69*, *70*), attributed to increased tail states arising from bandgap fluctuations and electrostatic disorder caused by compositional inhomogeneity, lattice strain, or charged defects (*70*, *71*). This redshift, observed in both CIGS and CuGaSe_2_ (CuGaSe_2_:Al,RbF), gradually recovers during or after DS. Previous studies linked this effect to the degree of Cu-deficient phase formation on the film surface (*69*). These results indicate that the “light-soaking effect” is fundamentally independent of the In/Ga difference; instead, off-stoichiometric disorder–related defects, such as substitutional alkali atoms on Cu sites, III_Cu_, defects, and their complexes, play the dominant role in governing the phenomenon.

**Fig. 5.**
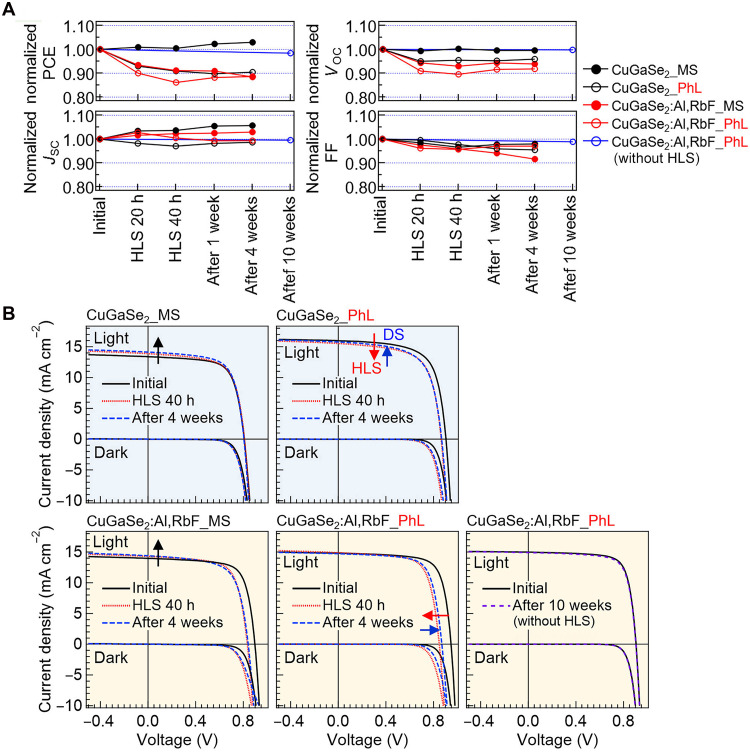
Variations in CuGaSe2-based device properties under HLS. (**A**) Relative changes in solar cell parameters. (**B**) Corresponding *J*-*V* curves under HLS at 90°C and after its cessation.

Figure 5 (A and B) shows the relative changes in the solar cell parameters and current-density–voltage (*J*-*V*) curves of CuGaSe_2_ and CuGaSe_2_:Al,RbF devices corresponding to Fig. 4 (A and B). Initial device parameters are listed in table S2. To corroborate the overall trend, box plots for three representative device types, MS-CuGaSe_2_, PhL-CuGaSe_2_, and PhL-CuGaSe_2_:Al,RbF, are shown in fig. S4 (A to C), which indicate that the trends in Fig. 5A are representative despite being based on single devices. The MS-CuGaSe_2_ device, which is initially lower performing, shows an apparent PCE improvement after HLS. However, this improvement is not due to an increase in *V*_OC_ and FF, as observed for light-soaking or bias-soaking effects in CIGS (*60*–*63*), but instead results from an increase in *J*_SC_ owing to a widened depletion width. In contrast, the other devices exhibit PCE degradation after HLS, primarily because of a decrease in *V*_OC_ and FF. For PhL-CuGaSe_2_, the reduction in *J*_SC_ is consistent with a narrower depletion width; although *N*_CV_ increases, *V*_OC_ decreases, leading to a lower PCE. Except for the MS-CuGaSe_2_ device, all other devices show degraded PCE after HLS. Some recovery occurs during DS, but not to the initial levels, and requires several weeks or longer. For several representative devices, variations are also monitored beyond four weeks; however, even after 10 weeks, the values do not fully return to their initial levels (fig. S4, A to C).

HLS at 50°C for 20 hours exhibit a similar degradation trend in CuGaSe_2_:Al,RbF (fig. S3B). These devices exhibit relatively rapid recovery; however, the PCE does not fully return to its initial levels within 4 weeks. Similarly, Kiselman *et al.* (*70*) reported degradation even in CIGS under HLS at approximately 50°C, with partial recovery after DS but incomplete return after more than 2 months (*70*). Ag-containing CIGS devices exhibited performance enhancement under the same HLS conditions (*70*), although degradation occurred during subsequent storage. Nevertheless, their performance remained higher than pre-HLS levels, which is beneficial for device operation.

In this study, most of the CuGaSe_2_-based devices exhibit degradation under HLS, although improvements have been reported for In-free CuGaSe_2_ devices under certain conditions (*19*). These observations demonstrate that the performance responses to HLS, enhancement, or degradation depend not solely on whether In or Ga is used but also on the intrinsic device properties and HLS conditions. Factors influencing the outcomes include defect characteristics in the photoabsorber, such as Cu deficiency (*69*), alkali content, compositional variation, and edge effects (MS or PhL). Further investigation is required to establish methods for controlling the device characteristics that reliably enhance or stabilize the performance upon HLS, in both CuGaSe_2_ and CIGS devices. In particular, the mitigation of *V*_OC_ loss remains critical, as decrements occur even when *N*_CV_ increases. It has been observed that applying HLS before cell separation enhances *V*_OC_ even in CuGaSe_2_ devices (*31*). This indicates that replacing MS with PhL is still insufficient and highlights the importance of developing effective cell-edge passivation strategies to maintain *V*_OC_ and ensure stable performance in CuGaSe_2_ devices.

### Further efficiency enhancement in high-*V*_OC_ CuGaSe_2_-based solar cells

Last, we report our recent progress and outlook on wide-bandgap chalcogenide solar cells. In earlier work, CuGaSe_2_-based devices reached PCEs above 12% with *V*_OC_ of ~0.95 V. Increasing the aluminum content raised *V*_OC_ to 1 V but degraded PCE below 12% (*21*). To overcome this trade-off, we explored strategies to improve *V*_OC_ without compromising the PCE.

CuGaSe_2_ devices exhibit a narrower depletion layer width than their CuInSe_2_ counterparts (*9*, *72*). Al incorporation further narrows the depletion width while increasing *N*_CV_ in both compounds (*20*, *21*).

**Fig. 6.**
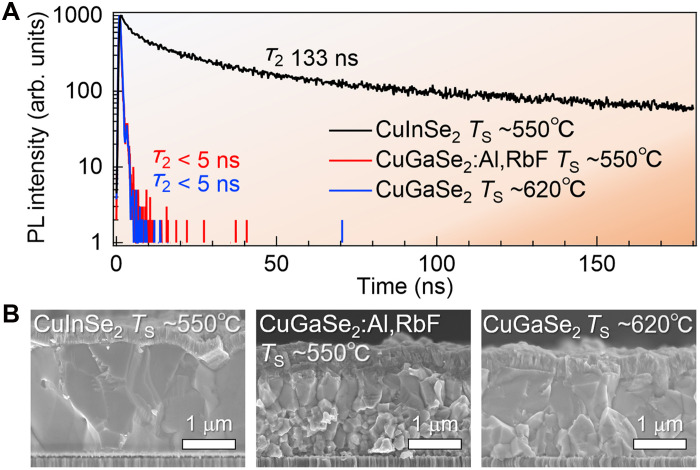
Time-resolved PL comparison between CuInSe_2_ and CuGaSe_2_. (**A**) Comparison of time-resolved PL transients measured on CdS-coated CuInSe_2_, CuGaSe_2_, and CuGaSe_2_:Al,RbF films. (**B**) Cross-sectional SEM images of the corresponding devices.

Carrier lifetimes in CuGaSe_2_ are generally shorter than in CuInSe_2_ or CIGS, primarily because of deep defect levels 0.5 to 0.8 eV below the CBM that act as strong bulk recombination centers (*51*, *73*). Photoluminescence (PL) lifetime measurements (Fig. 6A) highlight this difference, whereas SEM images (Fig. 6B) show that the CuGaSe_2_ films tend to have smaller grains compared with CuInSe_2_. Even when CuGaSe_2_ films with larger grains are grown at higher *T*_S_ (620°C), their PL lifetimes remain short, and Al/Rb incorporation does not provide significant improvement.

The narrow depletion width suggests that the region of a CuGaSe_2_:Al,RbF layer adjacent to the Mo back contact acts more as a hole-transport layer than as a photoabsorber. Therefore, a reduction in thickness is expected to reduce the undepleted bulk and suppress recombination.

**Fig. 7.**
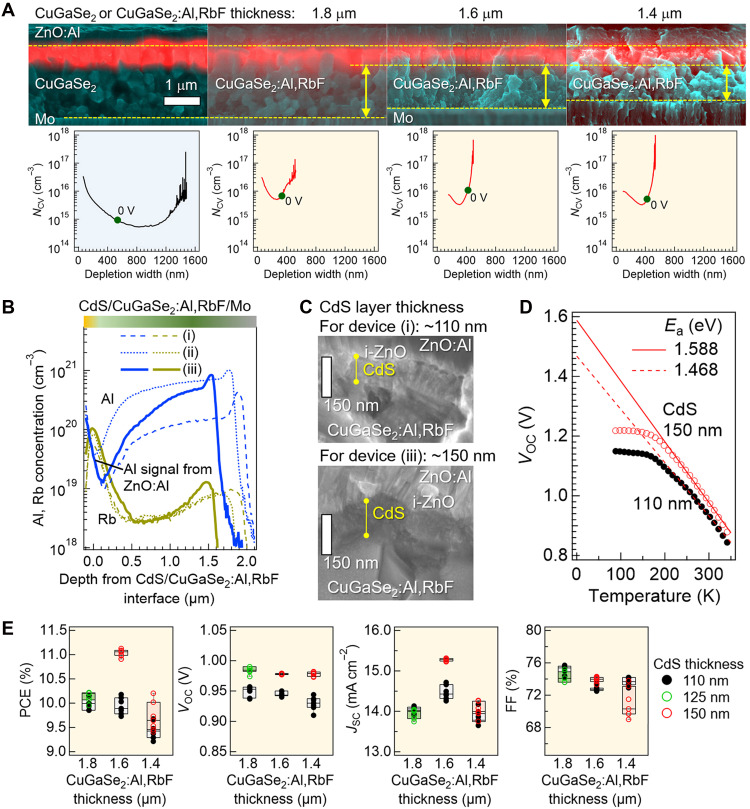
Device performance tuning through thickness and Al gradient engineering. (**A**) EBIC-SEM images of the CuGaSe_2_ and CuGaSe_2_:Al,RbF device cross sections and corresponding *N*_CV_-depletion width curves. (**B**) SIMS depth profiles of elemental Al and Rb in CuGaSe_2_:Al,RbF devices (i to iii). (**C**) Cross-sectional SEM images of the CdS layers in the corresponding devices. (**D**) Temperature-*V*_OC_ measurement results for devices with different CdS layer thicknesses. (**E**) Variations in solar cell parameters obtained from CuGaSe_2_:Al,RbF devices with various absorber thicknesses.

Figure 7A shows electron beam–induced current (EBIC)–SEM images for CuGaSe_2_ (1.8-μm thickness) and CuGaSe_2_:Al,RbF (1.8, 1.6, and 1.4 μm thick) devices. The *N*_CV_-depletion layer width curves of the corresponding devices are also shown. Here, the 1.8-μm-thick CuGaSe_2_ device serves as the reference. The 1.8-μm-thick CuGaSe_2_:Al,RbF device corresponds to the one reported in our previous study, which achieved a *V*_OC_ of 1 V (PCE: 11.7%) (*21*), and the 1.6- and 1.4-μm-thick CuGaSe_2_:Al,RbF devices were fabricated in this work. EBIC measurements indicate that the depletion layer width (red region) of the CuGaSe_2_ device exceeds 500 nm, becomes narrower with Al addition, and is consistent with the trend obtained from the *N*_CV_-depletion width curves. This trend agrees with the effect of Al addition observed in CuInSe_2_ devices (*20*). Reducing the CuGaSe_2_:Al,RbF absorber thickness does not change the depletion width but reduces the undepleted region, as expected. The 1.6-μm-thick (and the 1.4-μm-thick) CuGaSe_2_:Al,RbF devices are fabricated with a steeper Al-concentration gradient (device iii), as shown in Fig. 7B. Here, devices (i), (ii), and (iii) correspond to the previous record-PCE (12.25%) device, the high-*V*_OC_ (1.0 V) device from our previous study (*21*, *74*), and the 1.6-μm-thick device fabricated in the present work, respectively. The 1.4-μm-thick device was fabricated under the same conditions as the 1.6-μm-thick device, but with a shorter growth time to reduce the thickness. It can be seen that the Al concentration in device (iii) is as low as that in device (i) in the front-surface region and as high as that in device (ii) in the back-surface region. Furthermore, device (iii) uses a 150-nm-thick CdS buffer layer, which is thicker than the previously used 110 to 125 nm (Fig. 7C) because thicker CdS layers are expected to enhance *V*_OC_ in CuGaSe_2_ devices by reducing interface recombination. The use of a thicker CdS layer (150 nm) increases in the activation energy of recombination (*E*_a_), which is commonly regarded as an indicator of interfacial recombination (Fig. 7D). Note that Fig. 7D compares the *V*_OC_-temperature plots and extrapolated *E*_a_ values obtained from devices based on CuGaSe_2_:Al,RbF films grown in the same batch with different CdS thicknesses.

Although the performance of devices shown in Fig. 7A cannot be directly compared because of variations in Al content and gradient, several insights emerge from Fig. 7E. First, irrespective of the CuGaSe_2_:Al,RbF absorber thickness, higher PCEs are obtained with CdS layers thicker than 110 nm (125 or 150 nm), which is attributed to the enhancement in *V*_OC_. Second, 1.6-μm-thick devices (iii), featuring a steeper Al-gradient than 1.8-μm-thick devices (ii), yield higher *J*_SC_ while maintaining comparable *V*_OC_. Notably, both the 1.6- and 1.4-μm-thick devices deliver high *J*_SC_ despite incorporating relatively thick (150 nm) CdS layers, which typically induce parasitic absorption losses. The *J*_SC_ enhancement is attributed to a more effective BSF formed by the steep Al gradient, which reduces bulk-defect recombination (see fig. S5), as well as a lower Al concentration near the surface that narrows *E*_g_. Overall, combining thick CdS layers with steep Al gradients effectively suppresses both interfacial and bulk recombination, thereby improving photocurrent extraction.

The 1.4-μm-thick devices, however, exhibit degraded performance compared with the 1.8- and 1.6-μm-thick devices. This result is attributable to the relatively low absorption coefficient of CuGaSe_2_ compared with that of CuInSe_2_ (*75*). Although thinner layers minimize bulk recombination, excessive thinning reduces photon absorption. Hence, approximately 1.6 μm appears optimal for CuGaSe_2_:Al,RbF absorbers under current material properties.

Table 1 compares the solar cell parameters independently measured by the Photovoltaic Calibration, Standards and Measurement Team at the Renewable Energy Advanced Research Center, AIST, and Japan Electrical Safety and Environment Technology Laboratories (JET). An independently measured solar cell datasheet for device (iii) is shown in fig. S6.

**Table 1.** Benchmarks for 1.7 eV–bandgap chalcogenide solar cells. Solar cell parameters of three CuGaSe_2_:Al,RbF devices as measured by independent test centers.

Device	CdS thickness (nm)	PCE (%)	*V*_OC_ (V)	*J*_SC_ (mA cm^−2^)	FF (%)	Area (cm^2^)	Test center	Ref.
(i)	110	12.25	0.959	17.64	72.5	0.499	AIST	(*21*)
(ii)	125	11.7	1.007	15.62	74.5	0.5076	JET	(*21*)
(iii)	150	12.28	0.996	17.90	68.8	0.499	AIST	This study

Device (iii), developed from these insights, achieves a *V*_OC_ of 0.996 V, comparable to device (ii), while delivering a PCE of 12.28%, which matches or even exceeds that of device (i), the previous record. Although *V*_OC_ and *J*_SC_ improve, FF decreases, which is attributed to enhanced *J*_SC_ and corresponding *J*-*V* curve shape changes under stabilized measurements (25°C, continuous illumination, maximum power point tracking). This FF loss resembles the HLS-induced behavior, although *V*_OC_ does not degrade significantly, thus maintaining a high PCE. HLS at 50°C, however, results in a decrease in *J*_SC_ owing to a reduced depletion width, accompanied by an increase in *N*_CV_ (fig. S3B). These trends vary with the device composition, process fluctuations, and cell-edge effects. The detailed mechanisms remain unresolved yet are critical for advancing device performance.

In contrast to CuInSe_2_ and CIGS, CuGaSe_2_-based devices typically exhibited performance degradation under HLS. Nevertheless, as noted previously, performance improvements under HLS have also been reported for Ag-alloyed CuGaSe_2_ devices (*19*). Thus, the HLS-induced performance degradation observed in this study is neither intrinsic nor unavoidable. Further progress will require compositional tuning, incorporating stabilizing impurities, and cell-edge passivation. Ultimately, it is essential to suppress deep-level defects and develop growth techniques on transparent conductive substrates for tandem applications. Overcoming these challenges will enable CuGaSe_2_-based devices to achieve stable and high-efficiency performance for next-generation energy conversion.

## DISCUSSION

In this study, we examined wide-bandgap chalcogenide solar cells based on CuGaSe_2_/CdS heterojunctions. Interface analysis of a high-*V*_OC_ (1 V) CuGaSe_2_:Al,RbF device revealed no formation of Rb-Ga-Se phases at the interface, in contrast to the Rb-In-Se compounds often observed in In-containing devices, although Rb incorporation effectively enhances CuGaSe_2_:Al,RbF device performance. At the Mo/CuGaSe_2_:Al,RbF interface, strong EDX signals of Cu, Na, and O were detected, suggesting the presence of secondary phases comprising these elements. However, on the basis of the energy band diagram, the presence of Cu-Na-O compounds, such as Cu_2_O:Na, which are potentially formed from these elements, did not appear to impede carrier transport. *J*-*V* curves did not indicate hole-barrier formation at the rear interface. CuGaSe_2_-based devices exhibited behavior upon HLS that differed from typical CuInSe_2_-based devices, suggesting distinct metastable defect dynamics between Ga- and In-based compounds (metastable phenomena occur in both indium- and gallium-based chalcopyrites, but their behavior differs). For example, CuGaSe_2_ was more sensitive to thermal stress than CuInSe_2_. The use of a thicker CdS layer combined with a steep Al concentration gradient in the CuGaSe_2_ layer improved performance, which can be attributed to the reduction in recombination at both the interface and in the bulk. Incorporating these findings, we simultaneously achieved a high *V*_OC_ of approximately 1 V and a PCE of 12.28%, representing notably high performance among wide-bandgap (*E*_g_ ~ 1.7 eV) chalcogenide thin-film solar cells.

## MATERIALS AND METHODS

### Thin-film growth and solar cell device fabrication

CuGaSe_2_-based thin films were grown on Mo-coated SLG substrates using a three-stage coevaporation process (*41*). The thickness of the sputter-deposited Mo layer was 1 μm. The CuGaSe_2_-based film thickness was typically 1.8 to 2.0 μm; however, films with thicknesses of 1.4 and 1.6 μm were also grown to investigate thickness dependence in this study. By adjusting the growth time in the first stage, we controlled the resulting film thickness. The growth conditions for the CuGaSe_2_:Al,RbF films are detailed in our previous study (*21*). In brief, elemental Ga and Se were supplied during the first stage with *T*_S_ of 350°C. In the second stage, elemental Cu and Se were supplied, followed by the successive supply of Ga and Se in the third stage at a *T*_S_ of 550°C. Al and RbF were supplied simultaneously during approximately one-third of the first stage from the beginning. Additional RbF was provided during the third stage after the Cu/III ratio dropped below unity, as determined by pyrometer monitoring (*76*, *77*). The Cu/III ratios of as-grown films were estimated by EPMA measurements and were typically approximately 0.8 at the surface and 0.95 to 0.99 in the bulk, regardless of Al and RbF supply. A CdS buffer layer (110 to 150 nm thick) was deposited on the CuGaSe_2_:Al,RbF films by chemical bath deposition using an aqueous solution consisting of CdSO_4_, NH_2_CSNH_2_, aqueous ammonia, and water at 80°C. Intrinsic resistive ZnO (i-ZnO) and conductive Al-doped ZnO (ZnO:Al) layers with thicknesses of approximately 50 and 300 nm, respectively, were deposited by sputtering. A Ni/Al grid electrode was formed via electron-beam evaporation, and cell separation was performed by MS or photolithographic patterning, followed by etching (*64*). A thermally evaporated MgF_2_ ARC was used in these devices.

### Characterization of CuGaSe_2_:Al,RbF films and devices

Cross-sectional and surface observations of the devices were performed using SEM (HITACHI S-4800) at an acceleration voltage of 5 kV. Elemental depth profiles were obtained via SIMS measurements using Cs^+^ (5 kV, 66 μm by 66 μm) as the primary ion. The Al, Na, and Rb concentrations in the films were estimated from the secondary ion intensities using a calibration based on a known standard sample of ion-implanted material. Analytical TEM-EDX measurements were performed using a TEM equipped with an EDX detector (JEOL JEM-ARM300F2) operated at an acceleration voltage of 300 kV. EPMA (SHIMADZU C12132) measurements using acceleration voltages of 5 and 15 kV, which correspond to effective sampling depths of approximately 0.1 and 1 μm from the surface, respectively, were performed to estimate film compositions, such as Cu/III (*17*).

In-house measurements of solar cell parameters were performed under 100 mW cm^−2^ at 25°C (AM 1.5 G) illumination using a solar simulator (YAMASHITA DENSO ESS-1000) and dark conditions. The solar simulator was calibrated with a crystalline-Si standard cell; however, the resulting *J*_SC_ and PCE values were typically underestimated for wide-bandgap cells such as CuGaSe_2_. This discrepancy is considered to arise from factors such as spectral mismatch of the solar simulator light source. These values were used for relative comparisons, and precise values were provided by independently certified measurements. HLS was performed at 90°C under approximately 1-sun illumination (simulated light) in a nitrogen-purged box, whereas for some devices, the treatments were carried out under vacuum. No significant difference in the effect was observed between a nitrogen atmosphere and vacuum.

*C*-*V* measurements were performed using a 10-kHz AC signal sweeping from 0.6 to −3.0 V at room temperature (approximately 25°C), and the dielectric constant of 11.5ε_0_ was used for analysis, where ε_0_ is the dielectric constant of the vacuum. The *N*_CV_ values were calculated using the equation

$$N_{\text{CV}}\left(W\right)=\frac{2}{\left(q\;\varepsilon_{{\text{CuGaSe}}_{2}}\;\varepsilon_{0}\;S^{2}\left[d\left(1/C^{2}\right)/\mathrm{dV}\right]\right.}$$ (1)

where *W* is the depletion width and *S* is the device area.

The EQE curves were measured using AC mode (BUNKOUKEIKI CEP-25CI) with a white-light bias (JASCO XCS-150A xenon lamp) at 25°C. The time-resolved PL transient measurements were performed using an excitation light source at 532 nm, 0.90 to 5.52 mW, and a spot size of approximately 1 mm^2^ at room temperature (HAMAMATSU C12132). The PL decay was analyzed using the following biexponential function

$$I_{\text{PL}}\left(t\right)=C_{1}\text{exp}\left(-\frac{t}{\tau_{1}}\right)+C_{2}\text{exp}\left(-\frac{t}{\tau_{2}}\right)$$ (2)

where *I*_PL_(*t*) represents the PL intensity, *C*_1_ and *C*_2_ are coefficients, *t* is the time, and τ_1_ and τ_2_ are the decay times.

EBIC measurements were performed using an acceleration voltage of 15 kV with no bias voltage to compare the carrier diffusion length in CuGaSe_2_-based films with the depletion layer width obtained from *C*-*V* measurements. Temperature-*V*_OC_ measurements were performed over 80 to 340 K in vacuum under 100 mW cm^−2^ (AM 1.5 G) illumination. *E*_a_ values were obtained using the following equation

$$V_{\text{OC}}=\frac{E_{a}}{q}-\frac{\mathrm{AkT}}{q}\text{ln}\left(\frac{J_{00}}{J_{\text{SC}}}\right)$$ (3)

where *A*, *k*, *T*, and *J*_00_ are the diode ideality factor, Boltzmann constant, temperature, and recombination current density prefactor, respectively (*78*–*80*).
