# Supplementary material for: Bulk and interface engineering of 1.7 eV–bandgap chalcogenide solar cells enabling record efficiency
Source: Sci Adv. 2026 Mar 11;12(11):eaed4703. doi: 10.1126/sciadv.aed4703 (PMC12978232; doi:10.1126/sciadv.aed4703)
Supplement: Supplementary file 1 — Figs. S1 to S6 Tables S1 and S2 [file sciadv.aed4703_sm.pdf]

Science Advances
AAAS

Supplementary Materials for

Bulk and interface engineering of 1.7 eV–bandgap chalcogenide solar cells enabling record efficiency

Shogo Ishizuka and Noboru Taguchi

Corresponding author: Shogo Ishizuka, shogo-ishizuka@aist.go.jp

Sci. Adv. **12**, eaed4703 (2026)
DOI: 10.1126/sciadv.aed4703

This PDF file includes:

Figs. S1 to S6
Tables S1 and S2

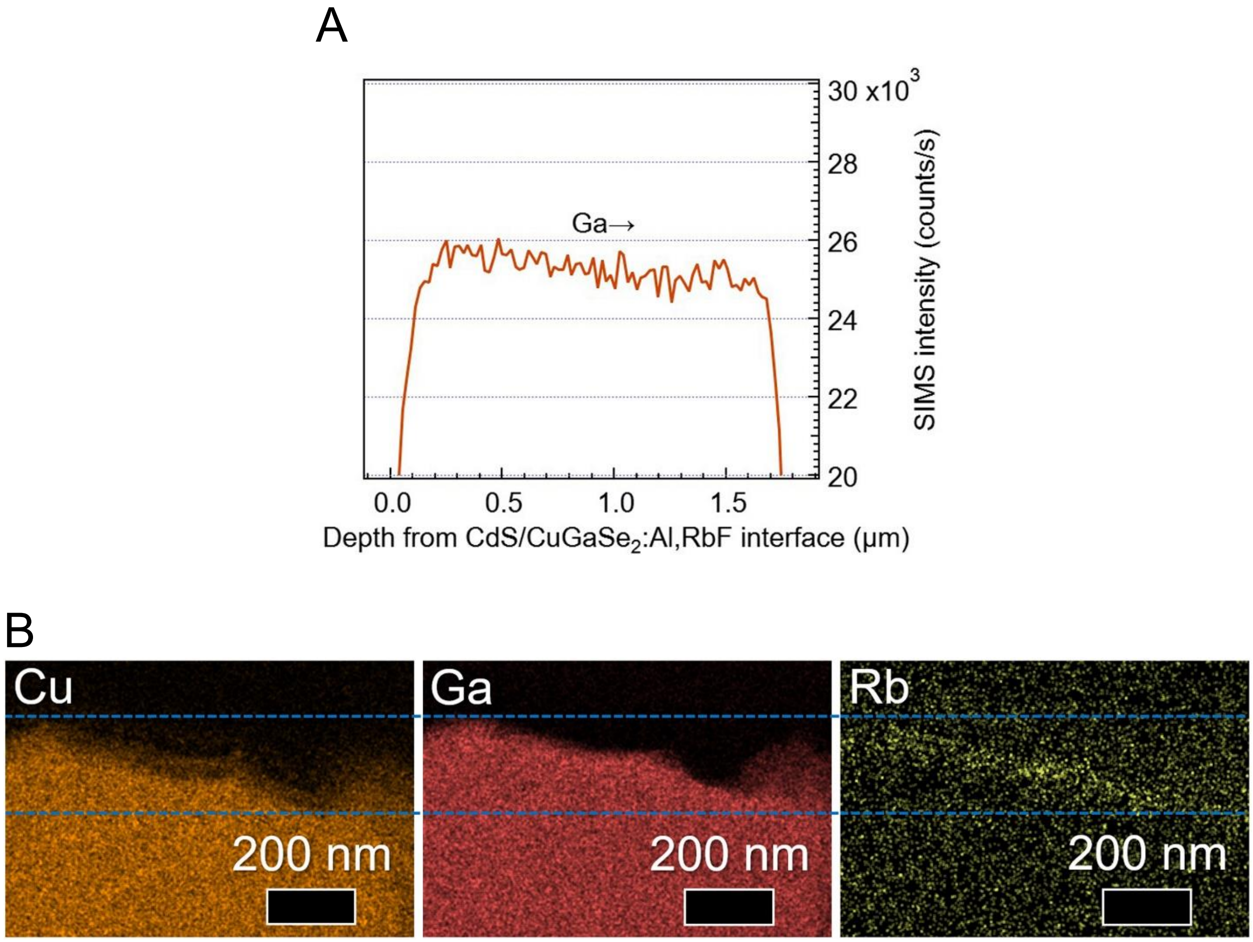

Fig. S1. Linear-scale Ga SIMS depth profile and EDX maps at the $CuGaSe_2$:Al,RbF/CdS interface. (A) Linear-scale Ga depth profile from Fig. 1B in the main text. A slight Ga gradient is observed in the opposite direction to that of Al. (B) Magnified EDX elemental maps of Cu, Ga, and Rb at the $CuGaSe_2$:Al,RbF/CdS interface, corresponding to the region shown in Fig. 1C of the main text. Blue dotted lines are provided as a guide to the eye.

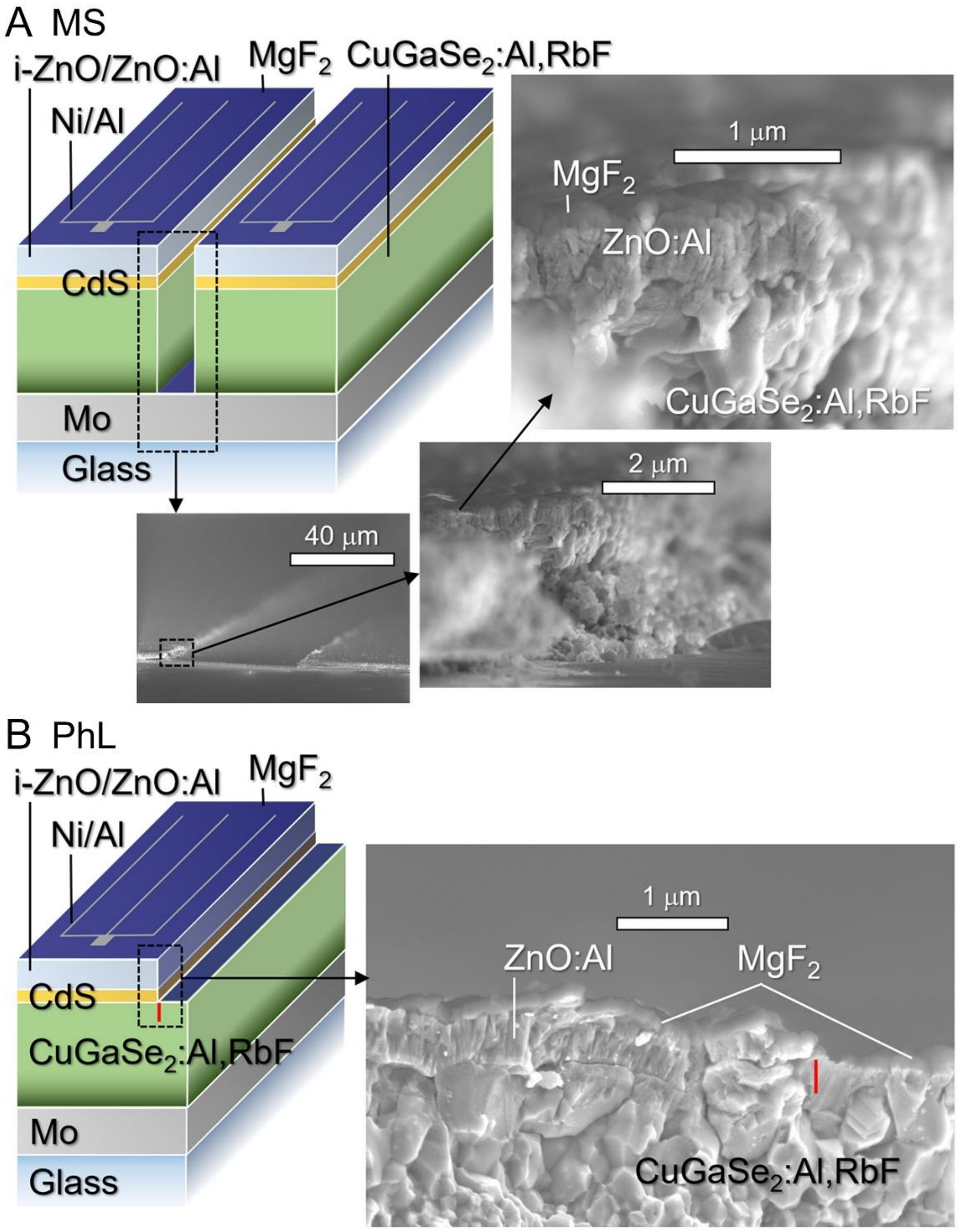

Fig. S2. Cell device edges. Schematic illustrations and cross-sectional SEM images of cell edges. (A) MS-processed devices. (B) PhL-processed device. Red lines indicate the cell edge.

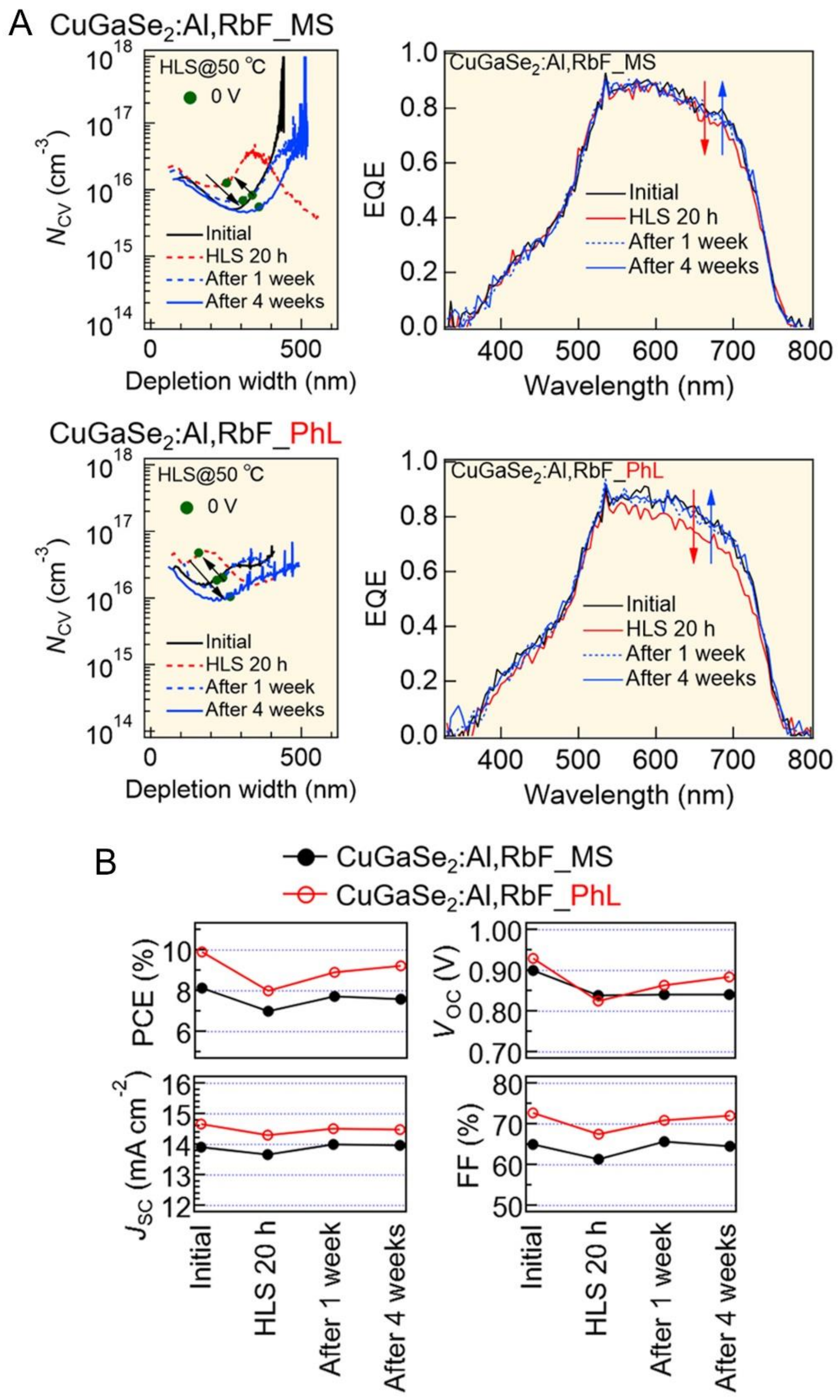

Fig. S3. Effects of HLS treatment performed at 50 °C. (A) Variations in the N_{CV}-depletion layer width curves and EQE curves upon HLS treatment performed at 50 °C. (B) Variations in solar cell parameters for the corresponding devices.

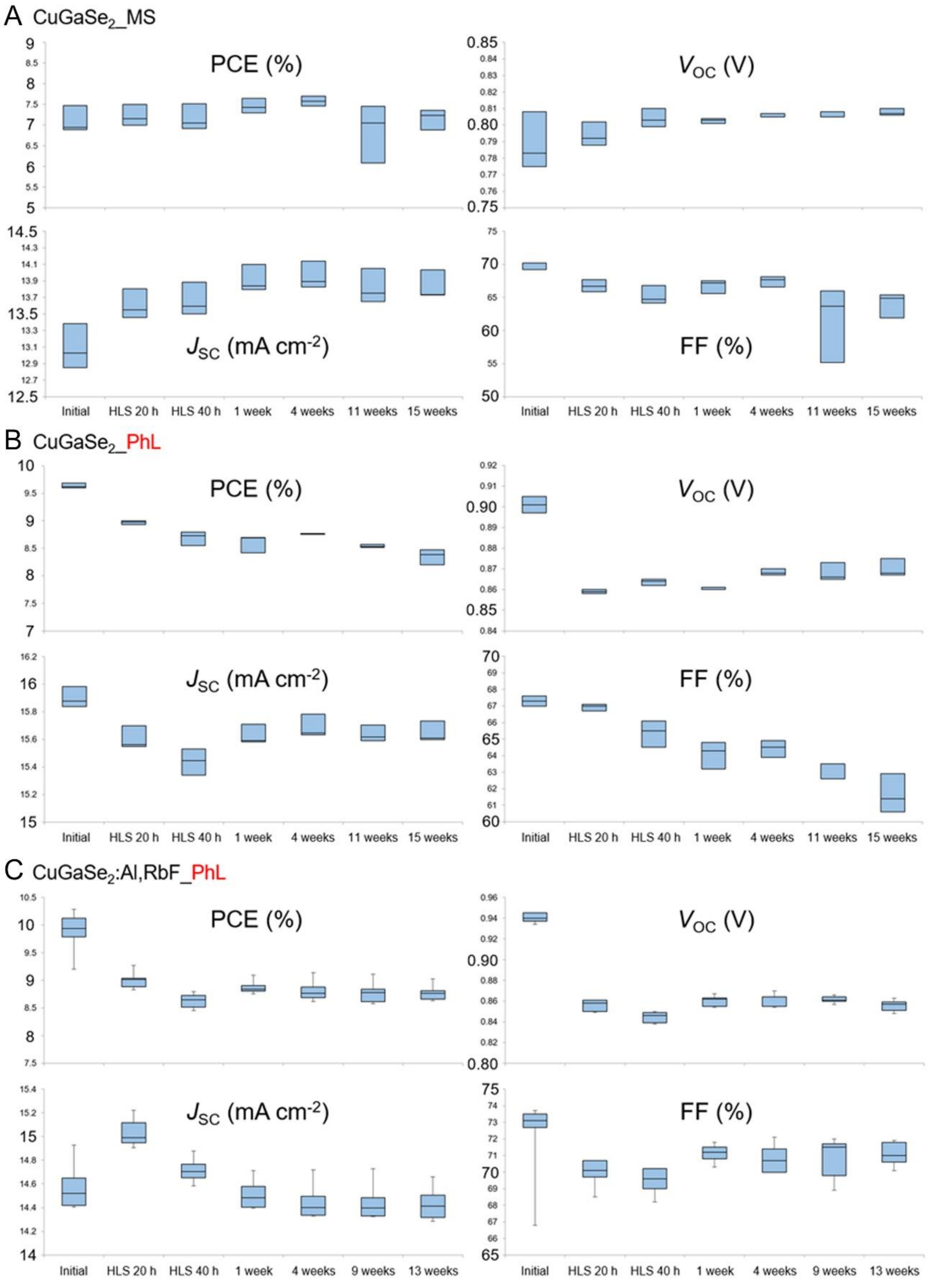

Fig. S4. Box plots showing variations in solar cell parameters. (A) MS-processed $CuGaSe_2$. (B) PhL-processed $CuGaSe_2$. (C) PhL-processed $CuGaSe_2$:Al,RbF devices. These devices are presented in Fig. 5A (main text).

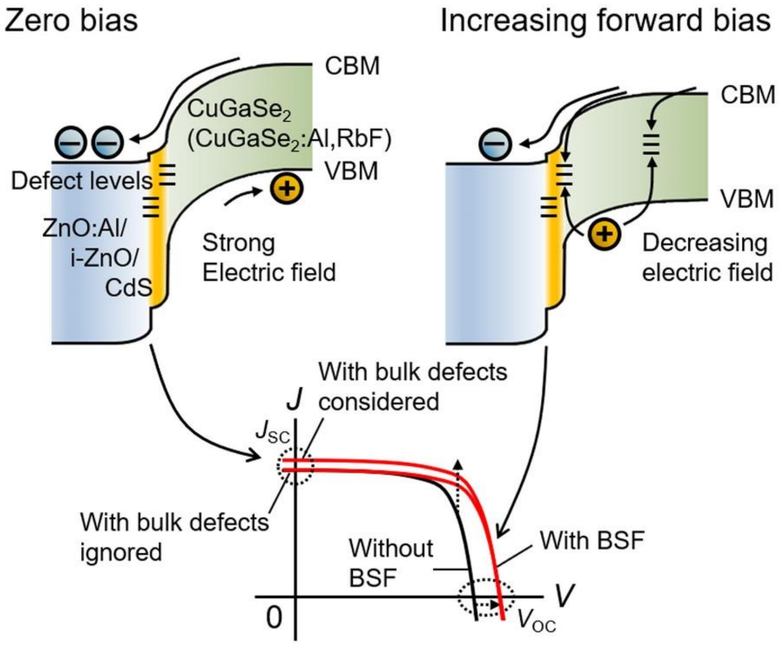

Fig. S5. Schematic energy-band diagrams and *J*−*V* curves. Schematic illustration of carrier transport at the $CuGaSe_2$ ($CuGaSe_2$:Al,RbF)/CdS interface under zero-bias and forward-bias conditions. The corresponding variations in the *J*−*V* characteristics for cases with and without a BSF and bulk-defect effects, is also shown.

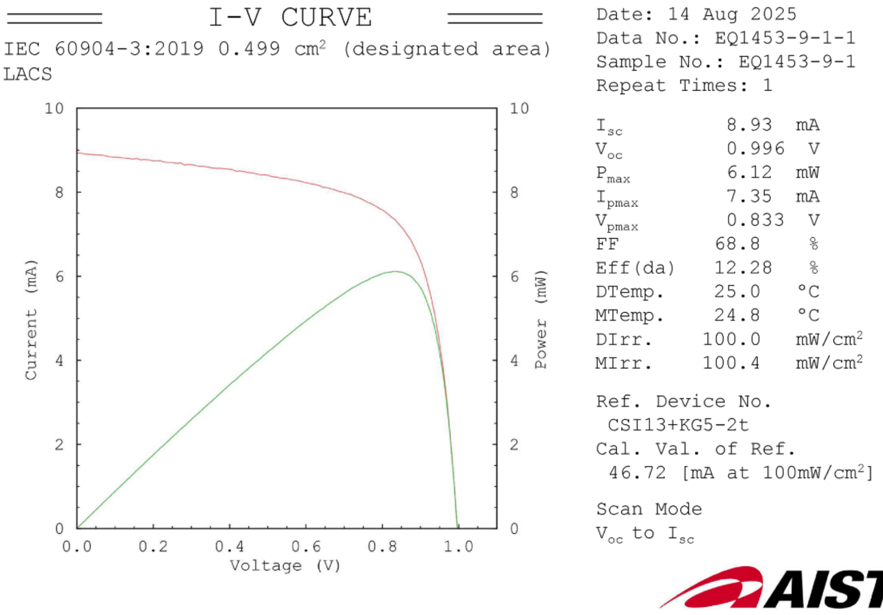

Fig. S6. Independently measured datasheet. A current-voltage and power-voltage datasheet obtained from device (iii) measured by the Photovoltaic Calibration, Standards and Measurement Team at the Renewable Energy Advanced Research Center, AIST.

Table S1. Diode parameters of MS- and PhL-processed $CuGaSe_2$:Al,RbF solar cells (Fig. 3B, main text) and representative $CuGaSe_2$ solar cells.

Cell separation	R_{SH} (Ω cm^2)	R_S (Ω cm^2)	A	J_0 (mA cm^{-2})
$CuGaSe_2$:Al,RbF				
MS (dark)	1467	0.51	2.08	1.2×10^{-6}
MS (1 sun)	869	0.55	2.22	6.5×10^{-6}
PhL (dark)	1.35×10^{5}	0.83	1.94	4.2×10^{-8}
PhL (1 sun)	2394	0.24	2.35	5.5×10^{-6}
$CuGaSe_2$				
MS (dark)	1544	0.56	2.12	1.1×10^{-6}
MS (1 sun)	903	0.40	2.48	2.6×10^{-5}
PhL (dark)	4.4×10^{5}	0.57	1.91	5.2×10^{-8}
PhL (1 sun)	1838	0.11	2.53	3.0×10^{-5}

Table S2. Solar cell parameters of $CuGaSe_2$ and $CuGaSe_2$:Al,RbF devices used in Fig. 5 (main text), measured in-house before HLS treatment.

Device	PCE (%)	V_{OC} (V)	J_{SC} (mA cm^{-2})	FF (%)
$CuGaSe_2$_MS	7.47	0.808	13.39	69.1
$CuGaSe_2$_PhL	9.68	0.905	15.98	67.0
$CuGaSe_2$:Al,RbF_MS	9.04	0.897	13.97	72.1
$CuGaSe_2$:Al,RbF_PhL	10.30	0.947	14.81	73.5
